# The Practitioner's Clinic

**Published:** 1888-02-11

**Authors:** 


### THE PRACTITIONER'S CLINIC.

BY A FAMILY PHYSICIAN.

**Concerning Congestion.**

THE term congestion is applied to conditions which are characterised by a more or less extensive accumulation of blood in any part of the body. To refer for one moment to the process of circulation, it will be remembered that the arteries, ending in arterioles or arterial capillaries, communicate with venous radicles or capillaries, which eventually form the veins; and that it is whilst passing through the capillary network that the processes of nutrition and oxidation of the tissues are effected; one of the most important results, in regard to the blood, being, that in this passage from arteries to veins it is deprived of a large proportion of its oxygen. Now, the two chief divisions of congestion are *active*, or the first stage of acute inflammation, previously described, and *passive*, due to causes not yet discussed. It may be laid down as a general principle that, whilst active congestion is a condition dependent upon abnormal processes connected with increased cardiac and arterial energy, passive congestion is dependent upon or associated with abnormal processes in the venous system and depressed cardiac energy. Indeed it may be asserted that active and passive congestion are respectively synonymous with arterial and venous congestion, although the capillaries of both systems may, and in all probability do, not unfrequently become secondarily involved. The effect of both on the blood vessels and the tissues in the vicinity is tension, indicated by pain. The tendency likewise is the same in both, a tendency to *stasis*, or complete stagnation of the blood, more pronounced by far in the arterial system, for the simple reason that it contains more fibrin. The causes of arterial congestion have already been discussed. Those of venous or passive congestion are cardiac debility or disease; impairment of power in the venous radicles; sympathetic irritation; obstructions in the course of the circulation, etc. It may result in complete stagnation, and when this occurs the blood in the affected part coagulates and forms what is known as a *thrombus*, the process of its formation being called *thrombosis*. The striking difference between the formation of such a clot, and that of the brawny diffuse swelling involving bloodvessels and surrounding tissues in acute inflammation, may be accounted for by something more than the mere impairment of vital power of the vascular walls; by the difference, namely, in cardiac and vascular energy observed during each process. In acute inflammation, the portion of capillary tissue involved by the *stasis*, is exposed to the full force of the excited circulatory energies, and the more fluid parts of the forming clot are driven through the capillary walls before actual coagulation occurs; whilst in venous stasis, coagulation goes on almost undisturbed, there being no undue excitement of the circulation at the point of formation. The exuded serum thus mingles with the blood on the proximal side of the clot, none of which, whilst it is forming, is driven by any distal force through the vascular wall.

Although, for facility in description, a hard and fast line has been now drawn between active and passive congestion, it is certain that there is sooner or later, more or less affection of both sets of capillaries in either form. The initial lesion, however, is either arterial or venous, and it is this which determines the character of the possible subsequent *stasis*. It is certain also that the existence of the one is very frequently the exciting cause of the development of the other; in the arterial form by the debility of the tissues, and the mechanically obstructive action of the effused inflammatory products; and in the venous by secondary irritation of the nervous system, leading to acute inflammation in the vicinity. Examples of the former class are seen in the passive congestion of the pharyngeal and laryngeal mucous membranes following upon acute chest affections, and the sympathetic congestion of the digestive tract so often concomitant, and indicated by furred tongue, dyspepsia, and inappetance. In the treatment of such conditions it is well to remember the advice of our old teachers—"Never give a tonic till you've got a clean tongue." The cleaning process may be rapidly and effectually carried out with alkalies, such as ammonia, soda and potash (nitrate or bi-carbonate), which act by giving greater flowing power to the blood, and thus prevent any tendency to stagnation; and judicious doses of such saline cathartics as the sulphate of magnesia, which, by unloading the capillaries of the intestinal tract, serve the same purpose in another direction. Alteratives such as the preparations of mercury and iodine are also frequently of great service. There is nothing of greater service in the chronic laryngeal catarrh of young children of from two to seven years, following upon acute bronchitis and pneumonia, than a dose of a grain to a grain and a-half of calomel mixed with sugar and given every second or third night for a week. An occasional mild "blue pill" with hyoscyamus serves the same purpose in adults. The action in all probability is "derivative" and "attractive," from the respiratory to the digestive tract, upon the energy of the circulation, and precisely similar in principle to that of a sinapism or blister applied externally for purposes of counter-irritation. Examples of the latter class may perhaps not be so frequently seen. I have observed cases in which acute pain of a spasmodic character has been complained of in the bowels, without rise of temperature, relieved for a time by pressure and friction, and the result of exhausting work with or without exposure to cold. The tongue has been moist and only slightly furred. I have seen such cases due, I believe, to simple passive congestion of the blood, from exhaustion in the mucous membrane and venous capillary system of the intestine generally, rapidly relieved by such remedies as ammonia, bicarbonate of soda, and brandy, with the application of sinapisms or turpentine stupes locally. But I have in other cases noted the gradual development of inflammatory mischief, in a rise of temperature and the characteristic dryness and hardness of the tongue. The stimulant action of brandy, the "liquefacient" one of ammonia, coupled with counter-irritation, generally suffice to effect a rapid cure in such cases. And now that we have so powerful an agent in dispersing stagnant blood, in the liquefacient *par excellence*, antipyrin, a dose of a scruple. sent half-an-hour before the doctor himself can go, will almost always suffice to enable the patient to greet him with a smile and grateful thanks for the "magic medicine" he has sent.

In some cases of this kind the delicacy of the intestinal canal is such that the very simplest articles of food cause more or less pain, and the slightest exposure to cold produces agonising spasms in one or other part of the tract. The tongue is generally moist, but coated with a varying amount of creamy fur, indicative of the passive congestion of the mucous membrane of the bowels. There is generally constipation. The treatment should be directed to unloading the capillaries with stimulant liquefacients and small doses of mild purgatives, combined with stomachic stimulants such as ginger, cardamoms, or capsicum, and sedatives like henbane.

Not unfrequently there is a sympathetic concomitant spasmodic affection of the gall bladder and cystic duct, indicated by pain in the right hypochondric region, darting through to the right shoulder blade. This should be treated on the same general principles, but with the additional prescription of a cholagogue and sedative in the shape of a calomel (gr. ij.) and opium (gr. ss.) pill (in place of the hyoscyamus) at bedtime.

*(To be continued.)*

